# Supplementary figures and images for: Sumoylation of the human histone H4 tail inhibits p300-mediated transcription by RNA polymerase II in cellular extracts
Source: eLife. 2021 Nov 8;10:e67952. doi: 10.7554/eLife.67952 (PMC8626089; doi:10.7554/eLife.67952)

Figure 1 – Source Data

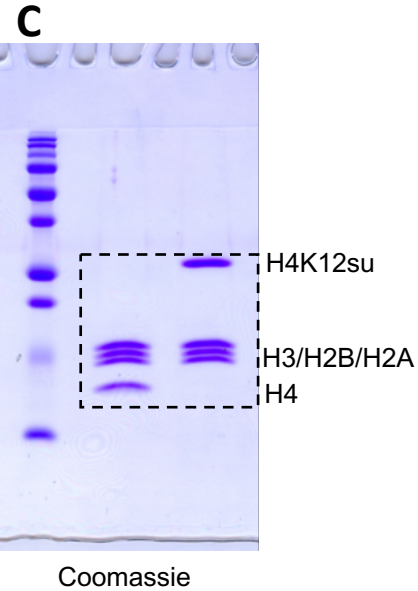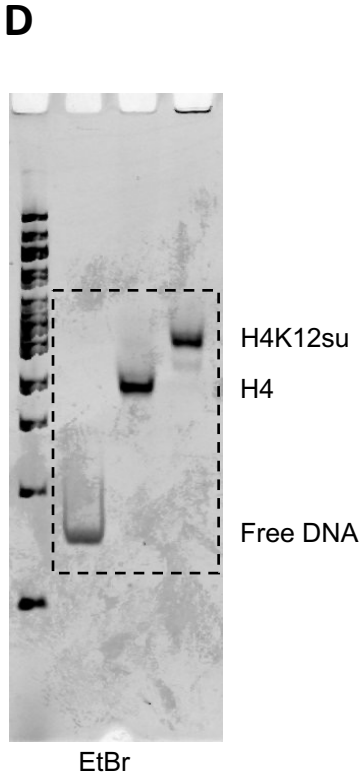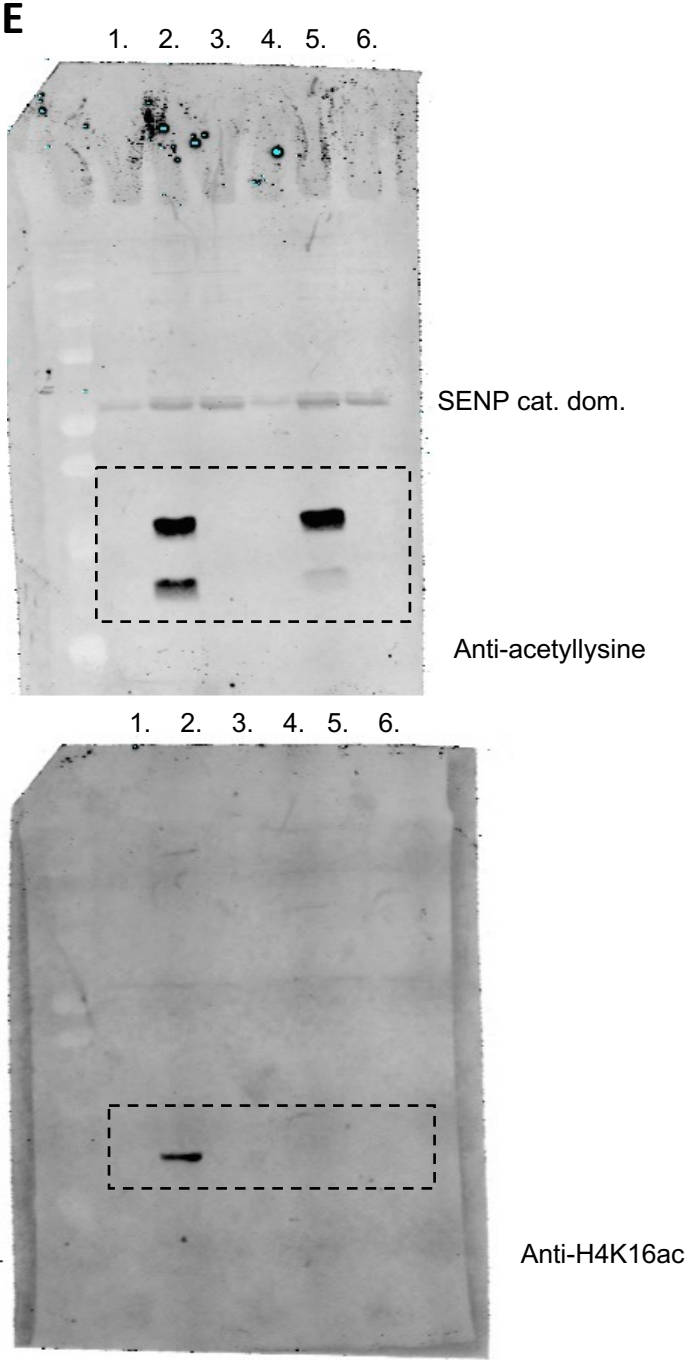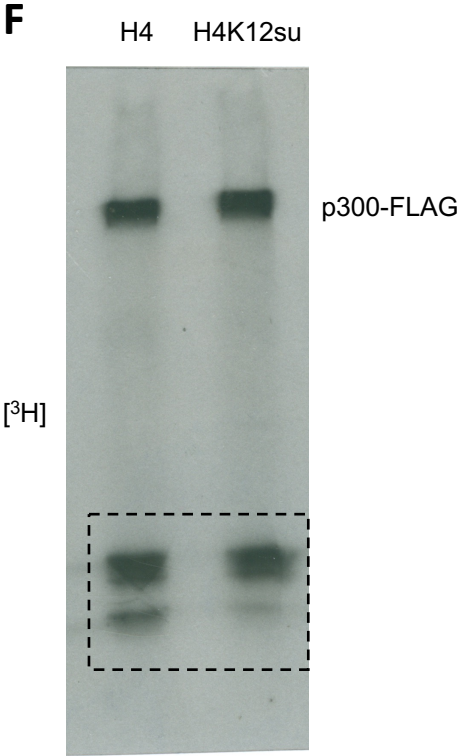

Supplement: Figure 1—source data 1. [file elife-67952-fig1-data1.pdf]

Figure 1, Figure Supplemental 1 – Source Data

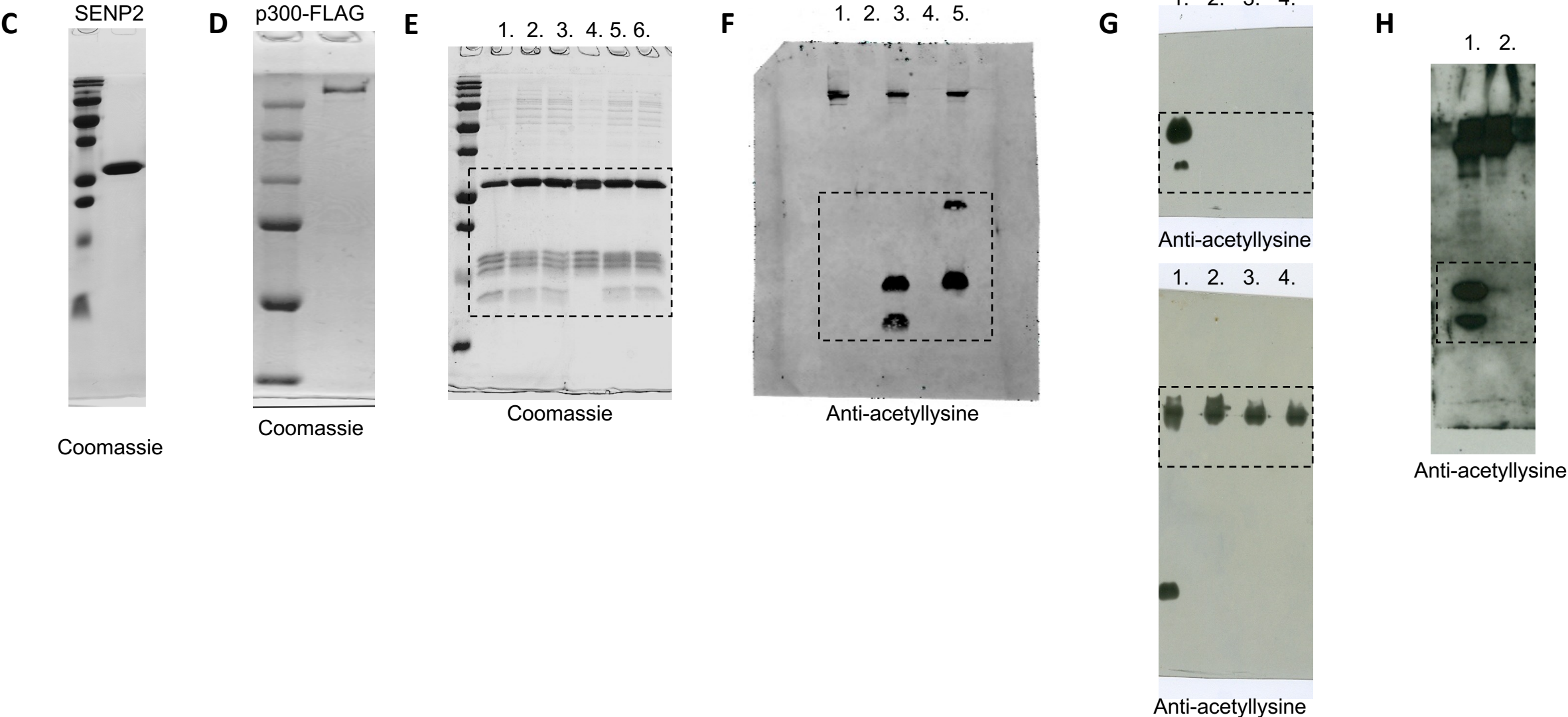

Supplement: Figure 1—figure supplement 1—source data 1. [file elife-67952-fig1-figsupp1-data1.pdf]

Figure 2 – Source Data

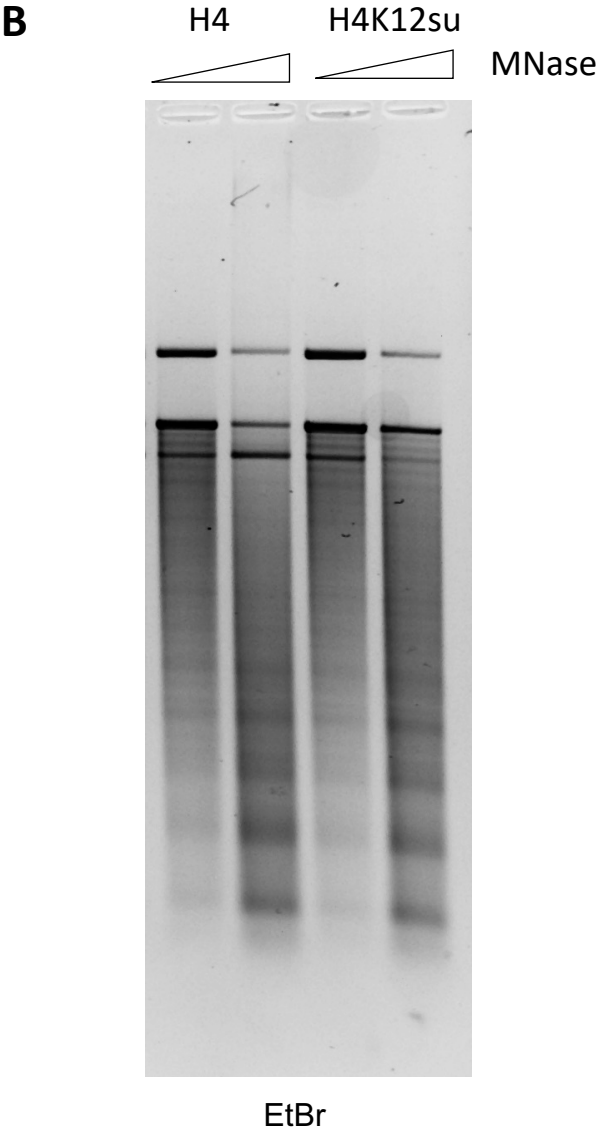

Supplement: Figure 2—source data 1. [file elife-67952-fig2-data1.pdf]

Figure 2, Figure Supplemental 1 – Source Data

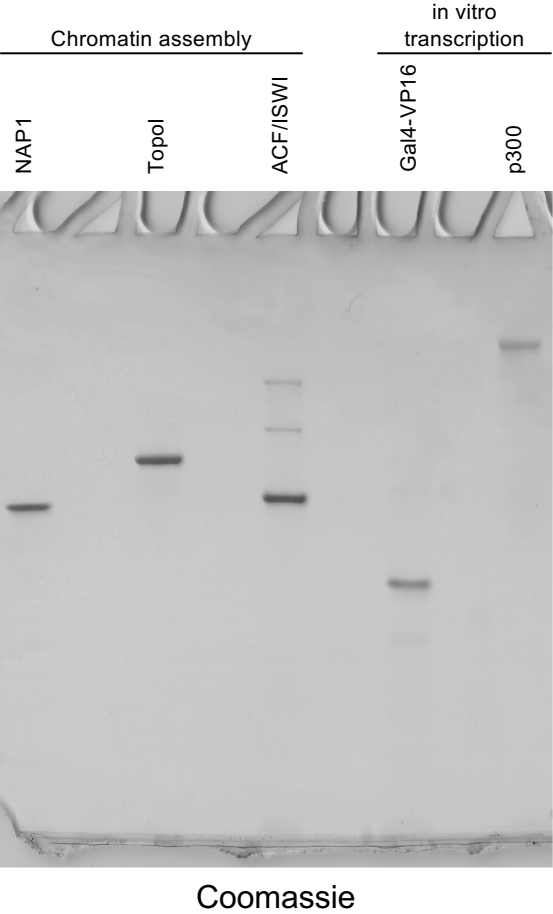

Supplement: Figure 2—figure supplement 1—source data 1. [file elife-67952-fig2-figsupp1-data1.pdf]

Figure 4 – Source Data

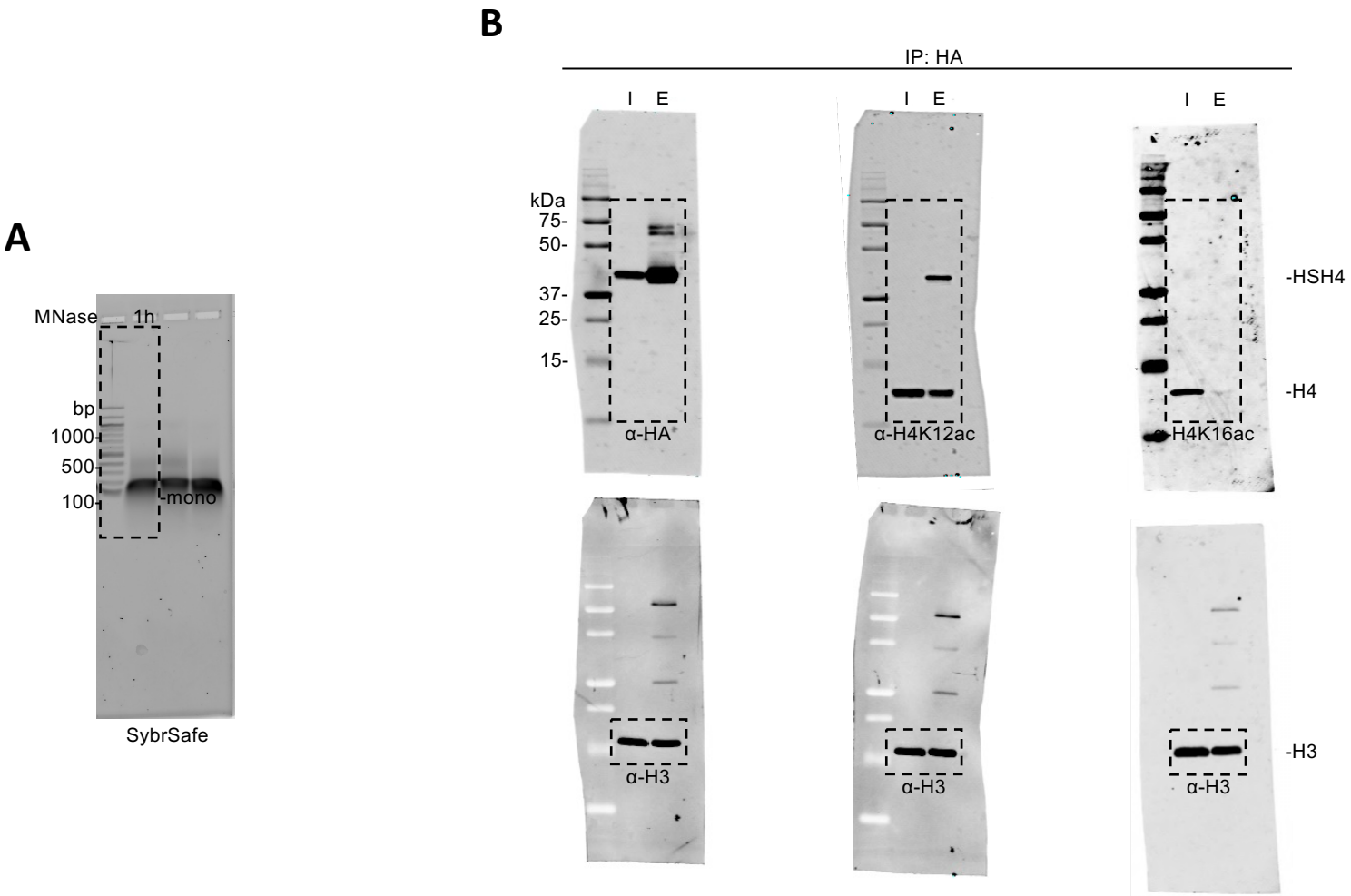

Supplement: Figure 4—source data 1. [file elife-67952-fig4-data1.pdf]

Figure 5 – Source Data

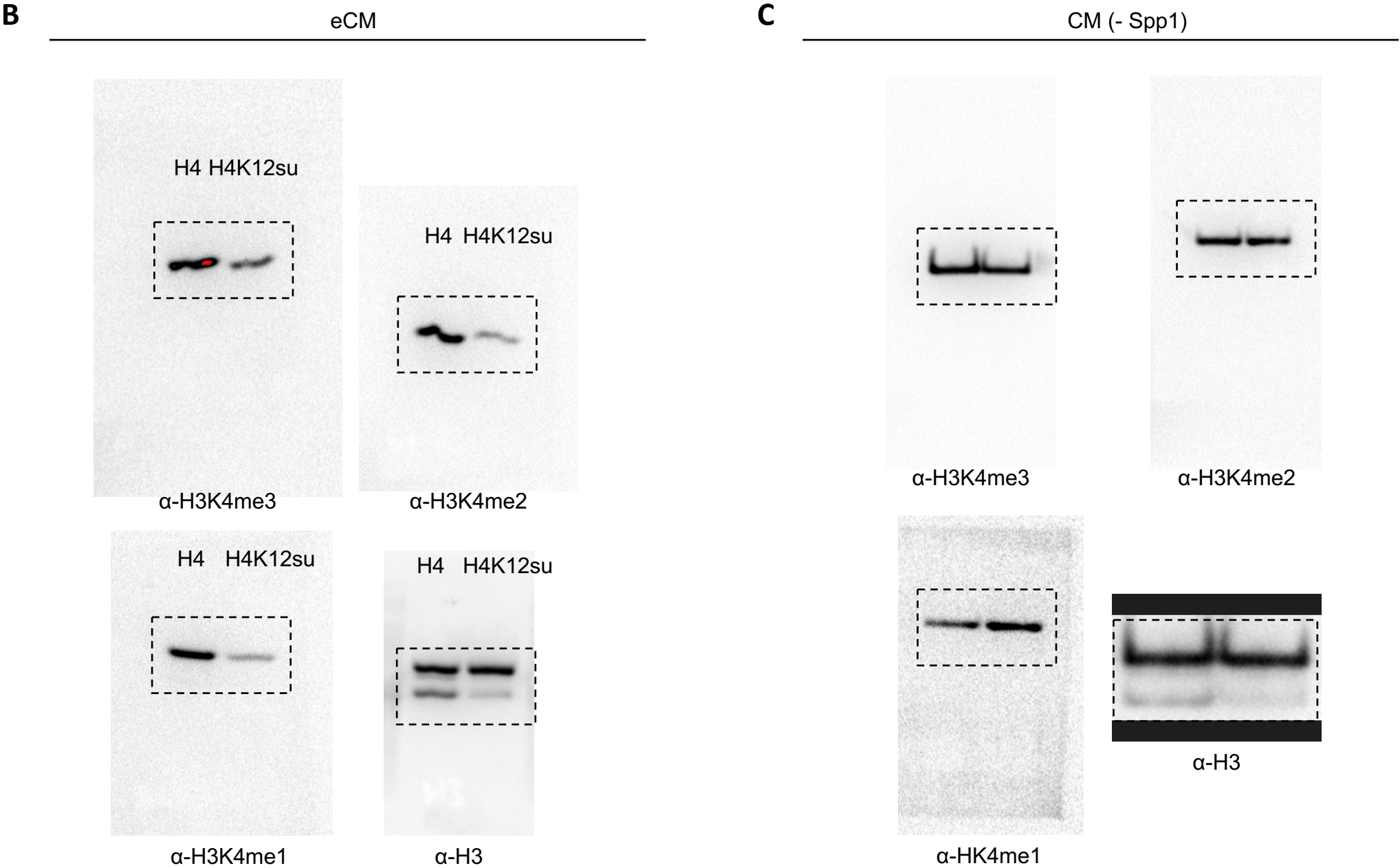

Supplement: Figure 5—source data 1. [file elife-67952-fig5-data1.pdf]

Figure 6 – Source Data

A

IP: HA

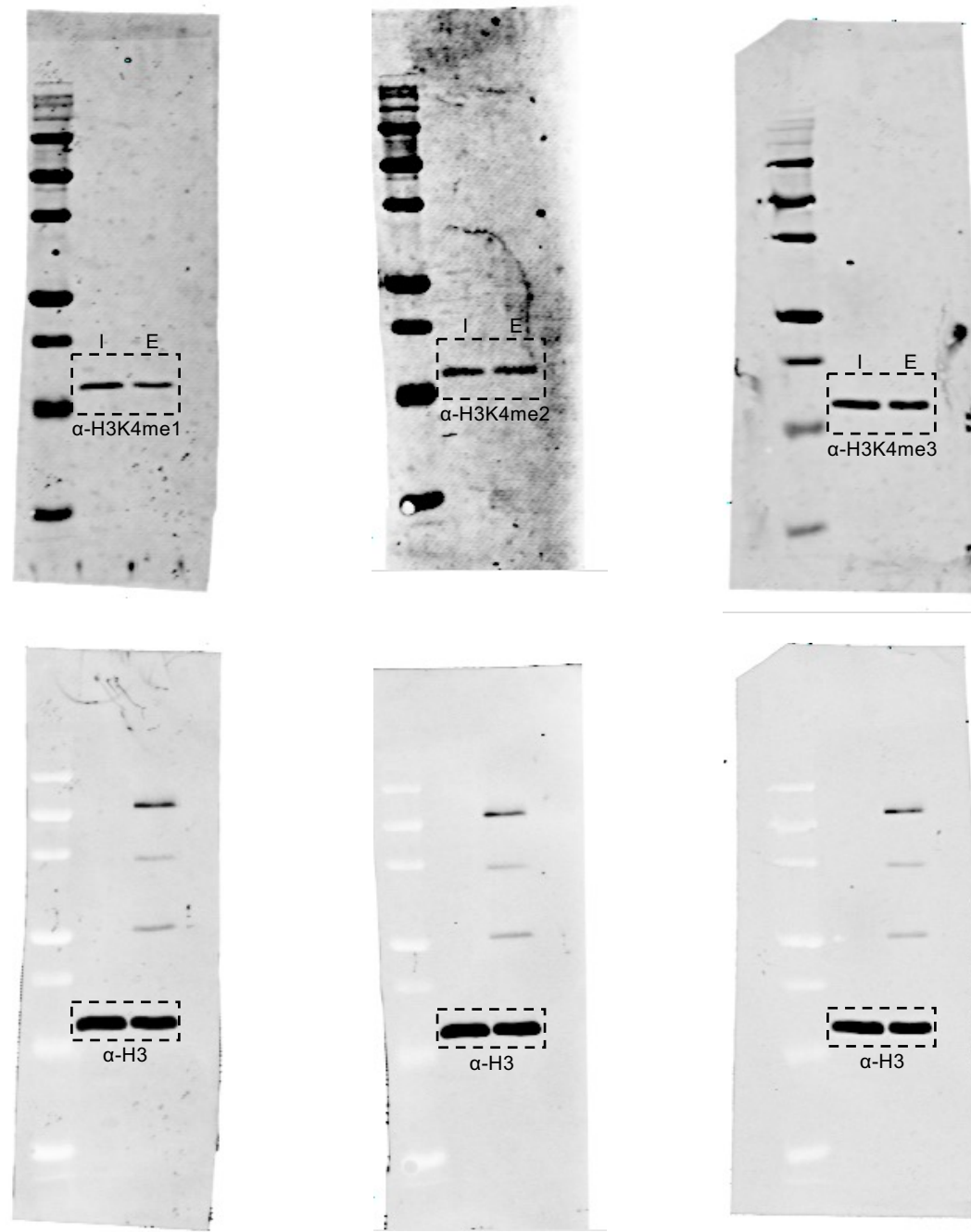

B

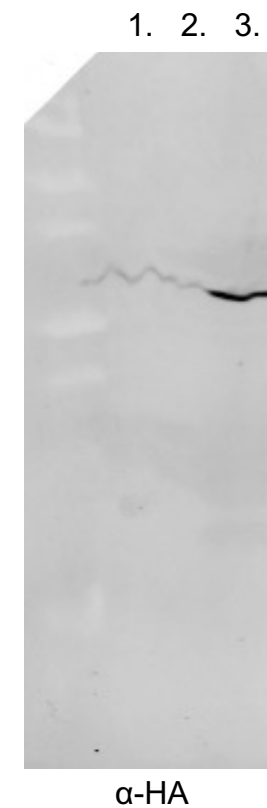

Supplement: Figure 6—source data 1. [file elife-67952-fig6-data1.pdf]
